# Deep Profiling of the Novel Intermediate-Size Noncoding RNAs in Intraerythrocytic *Plasmodium falciparum*

**DOI:** 10.1371/journal.pone.0092946

**Published:** 2014-04-08

**Authors:** Chunyan Wei, Tengfei Xiao, Peng Zhang, Zhensheng Wang, Xiaowei Chen, Lianhui Zhang, Meixue Yao, Runsheng Chen, Heng Wang

**Affiliations:** 1 Department of Microbiology and Parasitology, Institute of Basic Medical Sciences, Chinese Academy of Medical Sciences and School of Basic Medicine, Peking Union Medical College, Beijing, China; 2 Laboratory of Bioinformatics and Noncoding RNA, Institute of Biophysics, Chinese Academy of Sciences, Beijing, China; Bernhard Nocht Institute for Tropical Medicine, Germany

## Abstract

Intermediate-size noncoding RNAs (is-ncRNAs) have been shown to play important regulatory roles in the development of several eukaryotic organisms. However, they have not been thoroughly explored in *Plasmodium falciparum*, which is the most virulent malaria parasite infecting human being. By using Illumina/Solexa paired-end sequencing of an is-ncRNA-specific library, we performed a systematic identification of novel is-ncRNAs in intraerythrocytic *P. falciparum*, strain 3D7. A total of 1,198 novel is-ncRNA candidates, including antisense, intergenic, and intronic is-ncRNAs, were identified. Bioinformatics analyses showed that the intergenic is-ncRNAs were the least conserved among different *Plasmodium* species, and antisense is-ncRNAs were more conserved than their sense counterparts. Twenty-two novel snoRNAs were identified, and eight potential novel classes of *P. falciparum* is-ncRNAs were revealed by clustering analysis. The expression of randomly selected novel is-ncRNAs was confirmed by RT-PCR and northern blotting assays. An obvious different expressional profile of the novel is-ncRNA between the early and late intraerythrocytic developmental stages of the parasite was observed. The expression levels of the antisense RNAs correlated with those of their *cis*-encoded sense RNA counterparts, suggesting that these is-ncRNAs are involved in the regulation of gene expression of the parasite. In conclusion, we accomplished a deep profiling analysis of novel is-ncRNAs in *P. falciparum*, analysed the conservation and structural features of these novel is-ncRNAs, and revealed their differential expression patterns during the development of the parasite. These findings provide important information for further functional characterisation of novel is-ncRNAs during the development of *P. falciparum*.

## Introduction

It is evident that the vast majority of the DNA in eukaryotic genomes is transcribed into noncoding RNAs (ncRNAs) [1]. The ratio of non-protein-coding DNA to protein-coding DNA increases with organismal complexity. Such as the percentage of non-coding DNA is less than 25% in prokaryotes, approximately 50% in *Plasmodium* species and is as high as 98.5% in humans [2]. The recent ENCODE (Encyclopedia of DNA Elements) studies identified 9,640 long noncoding RNA loci in the human genome [3]. These results imply that ncRNAs play important roles in conferring developmental complexity. With the exception of certain well-established structural ncRNAs, such as rRNAs, tRNAs, snRNAs, and snoRNAs, regulatory ncRNAs have been demonstrated to play key roles in diverse biological processes. Over the last several decades, numerous regulatory ncRNAs were identified as important regulators of gene expression at the transcriptional, post-transcriptional, or translational levels *etc.* [4]–[8]. Among them, a large proportion are small RNAs approximately 20–30 nt in length (e.g., microRNAs, siRNAs, and piRNAs) or very large RNAs longer than 1 kb (e.g., Xist, HOTAIR, and other lincRNAs). However, intermediate-size ncRNAs (is-ncRNAs) [9], with lengths of 50–500 nt, have gained attention only recently. The limited published data suggest that is-ncRNAs play specific regulatory roles in the development of different organisms. For example, in *C. elegans*, they were found to be expressed in a highly stage-specific manner [9]; a fraction of is-ncRNAs are associated with transcription factor binding sites [10], and some is-ncRNAs are related to UV-induced DNA damage responses [11]. In silkworm (*Bombyx mori*), chicken (*Gallus gallus*) and rhesus monkey (*Macaca mulatta*), is-ncRNAs appear to play significant roles in lineage/species specification [12]–[14]. And in humans, is-ncRNAs were observed involving in the development and tumorigenesis of the fetal brain [15].

Malaria remains one of most severe infectious diseases worldwide, infecting an estimated 207 million people and causing 627,000 deaths annually (World Malaria Report 2013, WHO). The unicellular eukaryotic parasite *Plasmodium falciparum* (*P. falciparum*) is the most lethal causative agent of human malaria [16]. After the genome of *P. falciparum* 3D7 was sequenced in 2002 [17], more and more studies have tried to explore the precise mechanism of gene expressions including the regulation of it during the intraerythrocytic developmental stage, which is the only stage that is related to all clinical symptoms of the disease. Even so, until now, we are still far from fully understanding this parasite. Interestingly, although both siRNAs and miRNAs have been identified in some protozoan parasites, such as *T. gondii* and *G. intestinalis* [18], miRNAs have not yet been discovered in *P. falciparum* by traditional cloning and sequencing methods [19]–[21], and the Dicer and AGO genes are absent from the *P. falciparum* genome [21], [22]. These results imply that other unknown ncRNAs, such as is-ncRNAs, may constitute the noncoding transcriptome of *P. falciparum*. In 2009, Raabe *et al.* screened the *P. falciparum* genome for ncRNAs ranging in size from 10–500 nt using a cloning method and identified 298 is-ncRNAs among 630 ncRNAs from 25,000 cDNA clones [23]. Later, Broadbent *et al.* sought to investigate long noncoding RNAs longer than 200 nt in *P. falciparum* via a tiling microarray [24] and identified 64 is-ncRNAs. An additional 197 is-ncRNAs had been identified in other ncRNA studies based on comparative genomics analyses [25]–[27]. Considering that some *P. falciparum* DNA might be lost during the cloning process, and the 14 chromosomes of *P. falciparum* might not be fully covered by the probes in the tiling array study, we try to explore the whole profile of is-ncRNA in *P. falciparum* by using the RNA sequencing. We constructed an is-ncRNA specific library of intraerythrocytic *P. falciparum* 3D7 with rRNAs and U1–U6 RNAs depleted, and sequenced the library using Illumina/Solexa paired-end sequencing. After obtaining 1,198 novel is-ncRNA candidates, we analysed them for conservation and secondary structure classifications. Then, we used RT-PCR and northern blotting assays to confirm the expression of the molecules. The stage-specific expression patterns of a few randomly selected is-ncRNA candidates were detected by qRT-PCR. Our data further expands the understanding of the *P. falciparum* is-ncRNAs that are expressed during the erythrocyte stage, reveals the expression profiles of these ncRNAs, and suggests potential roles of these molecules in regulating the development of malaria parasites.

## Results

### Novel is-ncRNAs revealed by Illumina/Solexa paired-end sequencing

**Figure 1:**
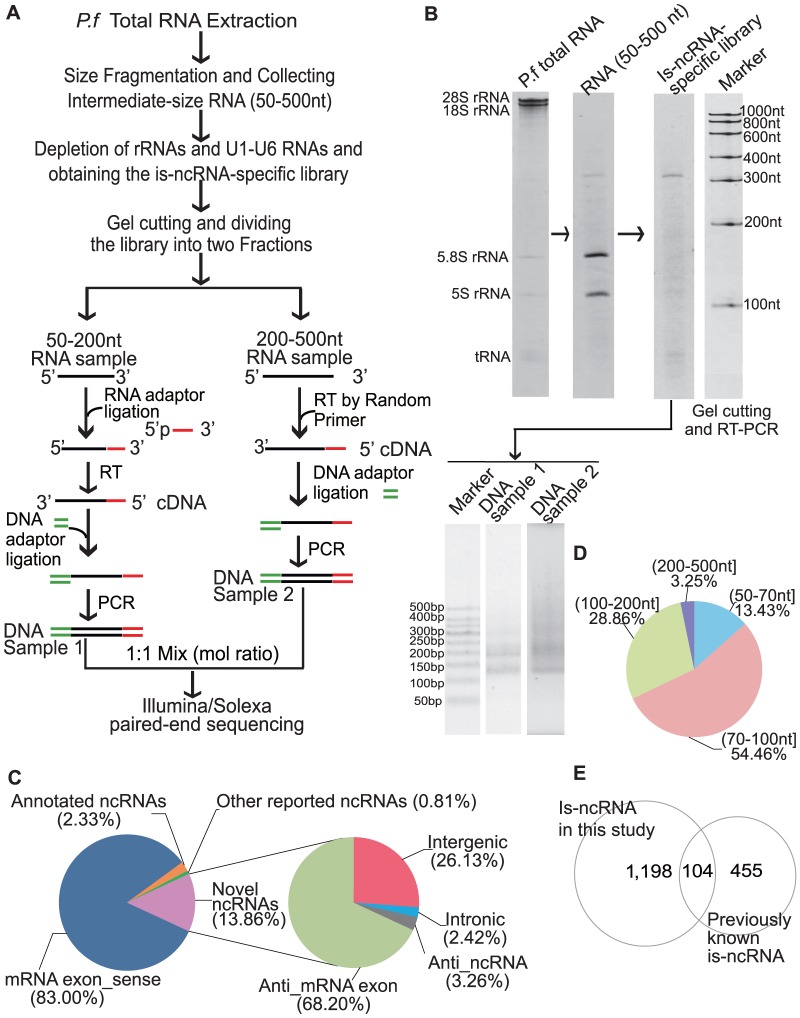
Illumina/Solexa paired-end sequencing and data analysis. (A) The strategy for the Illumina/Solexa paired-end sequencing library preparation. (B) 6% denaturing urea PAGE of RNA samples and 6% native PAGE of DNA samples for the Illumina/Solexa paired-end sequencing library preparation according to the flowchart shown in (A). (C) The genome location distribution of contigs assembled from the Illumina/Solexa paired-end sequencing reads. “Annotated ncRNA” represents ncRNAs in PlasmoDB 10.0, including rRNAs, tRNAs, snoRNAs, U1–U6 snRNAs, SRP RNA, RNAse P, and RNAse MRP RNAs. “Other reported studies” denotes the ncRNAs that were described in the five studies by Mourier T *et al.* (2008), Mishra PC *et al.* (2009), Raabe CA *et al.* (2009), Otto TD *et al.* (2010) and Broadbent KM *et al.* (2011) but not in PlasmoDB 10.0. (D) The size distribution of all the 1,198 novel is-ncRNAs. (E) All *P. falciparum* is-ncRNAs identified to date. “Previously known is-ncRNAs” denotes all the is-ncRNAs annotated in PlasmoDB 10.0 and reported is-ncRNAs in the five studies.

An is-ncRNA-specific library, specifically for identifying non-capped and non-polyadenylated ncRNAs, was constructed from the mixed intraerythrocytic stage of *P. falciparum* 3D7 (rings, trophozoites and schizonts) and subjected to Illumina/Solexa paired-end sequencing (Figure 1A and 1B). The sequencing yielded a total of 16,596,877 paired-end reads; 76.79% of the reads were mapped directly to the latest version of *P. falciparum* 3D7 genome (PlasmoDB 10.0) using Bowtie [28] (Materials and Methods). A total of 8,694 contigs were assembled from the qualified reads (Materials and Methods). Of these contigs, 7,216 (83.00%) overlapped with mRNA exons in the sense orientation; 203 (2.33%) overlapped with ncRNAs (annotated in PlasmoDB 10.0) in the sense orientation, covering 74.1% of the 141 is-ncRNAs in the PlasmoDB 10.0 and 80.9% of these ncRNAs if tRNAs, U1–U6 snRNAs, 5S and 5.8S rRNAs were excluded; and 70 contigs overlapped with ncRNAs that were reported in other five studies [23], [24], [26], [27], [29] but were not present in PlasmoDB 10.0. Compared with is-ncRNAs that have been reported in those five studies [23], [24], [26], [27], [29], these 70 contigs covered 5.7% of the 298 is-ncRNAs that were identified by the cloning method in 2009 [23], 4.7% of the 64 is-ncRNAs identified by tiling microarray analyses in 2011 [24], 2.5% of the 40 is-ncRNAs identified by high-scoring predictions and microarray analysis in 2008 [26], 5.6% of the 18 is-ncRNAs identified by computational and experimental approaches in 2009 [27], and none of the 27 polyadenylated is-ncRNAs detected by RNA-Seq in 2010 [29]. Besides all these contigs which were annotated as mentioned above, another 1,205 contigs were mapped to un-annotated locations of the genome, and were considered to represent novel putative ncRNAs. Among them, 1,198 contigs that ranged from 50 to 500 nt were considered as novel is-ncRNA candidates (Table S1 in File S1), including 817 anti_mRNA exon is-ncRNAs, 39 anti_ ncRNA is-ncRNAs, 313 intergenic is-ncRNAs, and 29 intronic is-ncRNAs (Figure 1C and Table 1). About 55% of these novel is-ncRNAs were in length between 70–100 nt, about 30% of them were in the size range 100–200 nt and is-ncRNAs ranged from 200–500 nt accounts for the smallest proportion (3.25%) (Figure 1D). Among these 1,198 novel is-ncRNAs, only six of them were represented by more than 50 reads, and 939 were represented by a single paired-end reads. These results indicate the effective enrichment of unknown is-ncRNAs with much lower expression levels. Thus, between the ncRNAs identified here and the 559 previously known is-ncRNAs that are in PlasmoDB 10.0 or have been reported in other studies, a total of 1,757 is-ncRNAs have been identified in *P. falciparum* to date (Figure 1E).

**Table 1:** Illumina/Solexa paired-end sequencing mapping to the *P. falciparum* genome and the distribution of assembled contigs.

Class	Number	Percent
Total paired-end reads	16,596,877	100%
Reads mapped to PlasmoDB 10.0	12,744,827	76.79%
Total assembled contigs	8,694	
Mapped to mRNA exon	7,216	83.00%
Mapped to annotated ncRNA in PlasmoDB 10.0	203	2.33%
Mapped to ncRNA reported in other studies	70	0.81%
Mapped to un-annotated locations (putative novel ncRNAs)	1,205	13.86%
Novel is-ncRNA candidates (50–500 nt)	1,198	
Anti_mRNA exon	817	68.20%
Anti_ncRNA	39	3.26%
Intergenic	313	26.13%
Intronic	29	2.42%

### Conservation analysis of the novel is-ncRNAs

**Figure 2:**
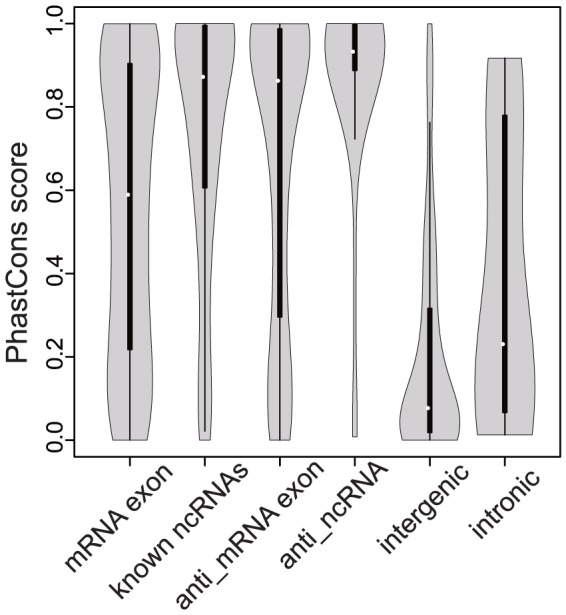
Conservation analysis of the novel is-ncRNAs. Conservation analysis of is-ncRNAs based on the phastCons score distributions across eight *Plasmodium* species: *P. falciparum, P. reichenowi*, *P. gallinaceum*, *P. vivax, P. knowlesi, P. yoelii*, *P. berghei*, and *P. chabaudi*. The white dots indicate the median phastCons scores of the RNAs in each class, and the bold black lines indicate the interquartile range.

The conservation of the novel is-ncRNA candidates across different *Plasmodium* species was analysed using phastCons scores [30]. The phastCons scores were available for 1,033 of the 1,198 novel is-ncRNA candidates (Table S1 in File S1, Material and Methods). Of the 1,033 is-ncRNAs, 57% (589) displayed average PhastCons scores ≥0.5 compared to *P. reichenowi, P. gallinaceum, P. vivax, P. knowlesi, P. chabaudi, P. berghei*, and *P. yoelii*. Novel anti_mRNA exon is-ncRNAs and anti_ncRNA is-ncRNAs were more conserved than the mRNA exons and known ncRNAs, respectively (Wilcoxon test, *P*-value<0.01). Among the novel is-ncRNAs, the anti_ncRNA is-ncRNAs were the most conserved and 87.5% of them had average phastCons scores>0.5. The intergenic and intronic is-ncRNAs were much less conserved (P<0.01) than the RNAs from other loci, and nearly 70% of the intergenic is-ncRNAs had average phastCons scores≤0.2 (Figure 2, Table S1 in file S1). This result may contribute to the elucidation of the evolutionary divergence of the genus *Plasmodium*.

### Structure analysis of the novel is-ncRNAs

To identify whether the novel intergenic and intronic is-ncRNAs belong to known functional classes of ncRNAs, the 313 intergenic and 29 intronic is-ncRNAs were analysed by snoGPS [31] and snoReport [32]. A total of 22 is-ncRNAs with clear snoRNA characteristics were identified (Table S2 in file S1). Of these is-ncRNAs, 20 were identified as H/ACA box snoRNA candidates, one was identified as a C/D box snoRNA candidate, and one (nc-594) was probable a small Cajal body-specific RNA candidate which showed both H/ACA and C/D box characteristics. A comparison to the 1,371 ncRNA families in the Rfam database [33] revealed no additional potential homologs of known ncRNAs among the 313 intergenic and 29 intronic is-ncRNAs.

**Figure 3:**
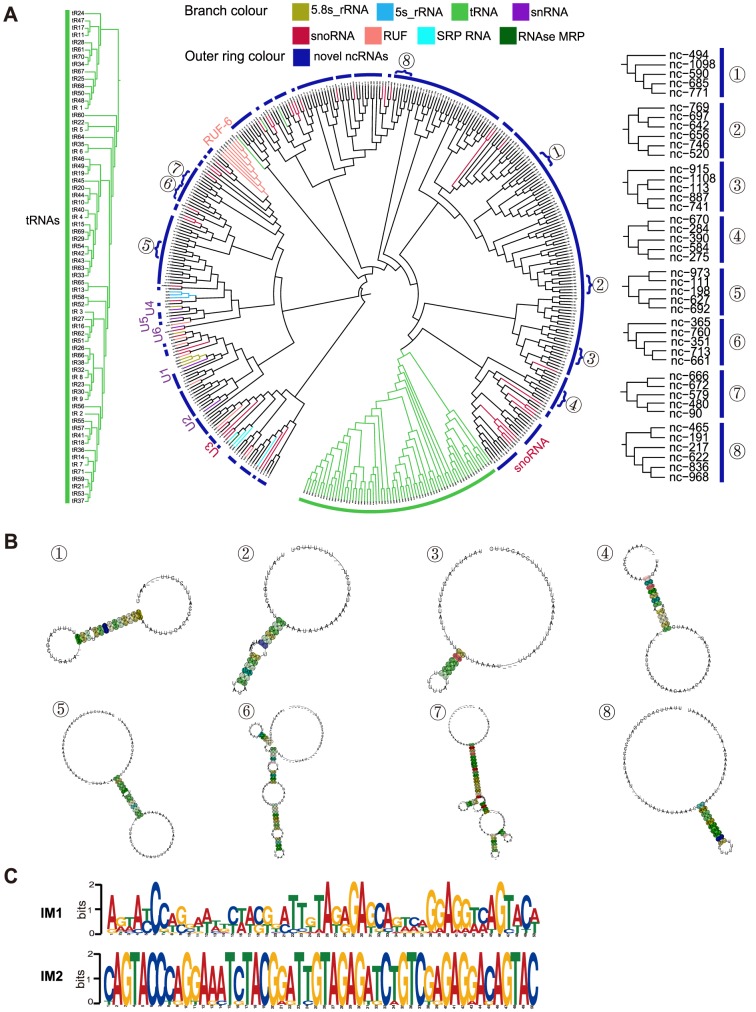
Structure-based analysis of the novel intergenic is-ncRNAs. (A) Clustering of the 313 novel intergenic is-ncRNAs and 141 known functional ncRNAs with intermediate sizes based on their predicted secondary structures. The blue outer ring segments indicate the novel is-ncRNAs, and the different branch colours indicate different types of known functional is-ncRNAs. The blue brackets indicate the eight potentially novel is-ncRNA classes, which are shown in a greater level of detail at the right side of the circular tree, and the green out ring indicates the tRNA cluster, which is shown in a greater level of detail at the left side of the circular tree. (B) Predicted secondary structures of the eight potentially novel is-ncRNA classes in *P. falciparum*. (C) Internal motifs (IM1 and IM2) of the novel intergenic is-ncRNAs beyond the eight novel classes.

Further, because a lack of primary sequence conservation does not necessarily signify an absence of function [34], and because a large class of functional ncRNAs possess characteristic secondary structures that are well conserved over evolutionary timescales [35], we characterised the 313 intergenic is-ncRNAs using the LocARNA-based clustering method [36], which was used previously to identify novel ncRNA classes in *C. elegans* [10]. A set of 141 known is-ncRNAs in PlasmoDB 10.0 was also subjected to this clustering analysis. Structural ncRNAs, such as tRNAs, snoRNAs, and other RUFs (RNAs of unknown function), were efficiently clustered into distinct classes, and several snoRNA candidates predicted by snoGPS and snoReport were also clustered (Figure 3A). In addition, using this clustering method, eight tightly knit clusters (including 42 is-ncRNAs, Table S2 in file S1) including at least five is-ncRNAs were identified, representing eight potential novel ncRNA categories (Figure 3A). The is-ncRNAs in novel class 1 are between 68 and 72 nt in length. Those in novel classes 2–4 and 8 range from 69 to 90 nt, and those in novel class 5–7 are between 104 and 151 nt in length (Table S1 and S2 in file S1). The is-ncRNAs in novel classes 1,3–5 and 8 contained two structural loops and one stem, those in novel class 2 contained three structural loops and two stems while those in novel classes 6 and 7 were the most complicated, with more than five structural loops and five stems (Figure 3B).

A motif analysis was performed on the remaining 271 intergenic is-ncRNAs. This analysis revealed two internal motifs (IM1 and IM2, Figure 3C) that were shared by 18 and 9 is-ncRNAs, respectively (Table S3 in file S1). IM1 and IM2 were similar to serine/arginine-rich splicing factor 1 binding RNA motif and CCR4-NOT transcription complex subunit 4 binding RNA motif in *Homo sapiens*, respectively (P<0.01) (Figure S1 in file S2). These findings may facilitate the prediction of targets of these is-ncRNAs.

### Experimental confirmation of the novel is-ncRNAs

**Figure 4:**
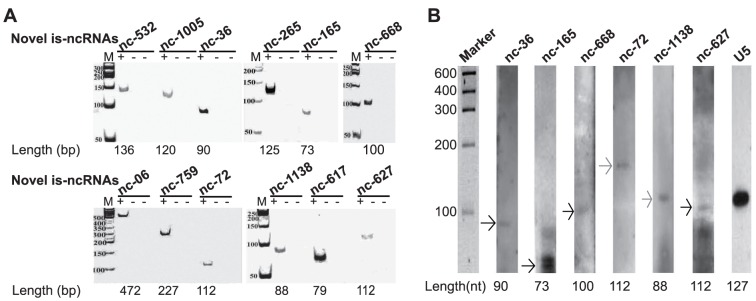
Experimental validation of novel is-ncRNAs. (A) RT-PCR confirmation of the novel is-ncRNAs. Twelve of the is-ncRNAs are shown as examples, and the remaining 48 are documented in Figure S1 in file S2. Each is-ncRNA is shown in three adjacent lanes, from left to right: DNase-treated RNA RT-PCR (“+”), RT-PCR with no RNA template (left, “−”, negative control), and RT without reverse transcriptase (right, “−”, negative control). “M” indicates the 50 bp DNA ladder. “Length” indicates the expected size of the is-ncRNAs based on the Illumina/Solexa paired-end sequencing assembly data. (B) Northern blotting validation of six novel is-ncRNAs. U5 snRNA was used as an internal control. The arrows indicate the sizes of the is-ncRNAs; the black arrows indicate products with lengths consistent with those of the RT-PCR products, and the grey arrows indicate products with lengths that were larger than those determined by RT-PCR. “Length” indicates the expected size of the is-ncRNA based on the Illumina/Solexa paired-end sequencing assembly data and RT-PCR.

To test the expression of the novel is-ncRNAs, 78 randomly selected novel is-ncRNA candidates were subjected to reverse transcription polymerase chain reaction (RT-PCR). Sixty of these tested novel is-ncRNA candidates (Table S4 in file S1), accounting for 76.9%, were amplified a fragment of the expected size (Figure 4A, Figure S2 in file S2). Among the 60 RT-PCR products, nine randomly selected ones were subjected to Sanger sequencing, and all of the sequences were consistent with the contigs assembled from the Illumina/Solexa paired-end sequencing, confirming the results of the Illumina/Solexa paired-end sequencing analysis. Further, among the RT-PCR confirmed is-ncRNAs, six randomly chosen candidates were validated by northern blotting assay. All of the results indicated the presence of a transcript in the expected intermediate-size range (Figure 4B). For four of the transcripts, the determined lengths were consistent with the Illumina/Solexa paired-end sequencing and RT-PCR. For the remaining two candidates (nc-72 and nc-1138), their size revealed by the northern blotting assay were larger than that obtained by RT-PCR, indicating that the RT-PCR analysis did not identify the full-length transcripts. This observation suggests that only portions of the primary transcripts were assembled. The signal intensity of the is-ncRNAs was much weaker than that of the U5 snRNA, further demonstrating the low expression levels of the novel is-ncRNAs.

### Expression profiles analysis of the novel is-ncRNAs

**Figure 5:**
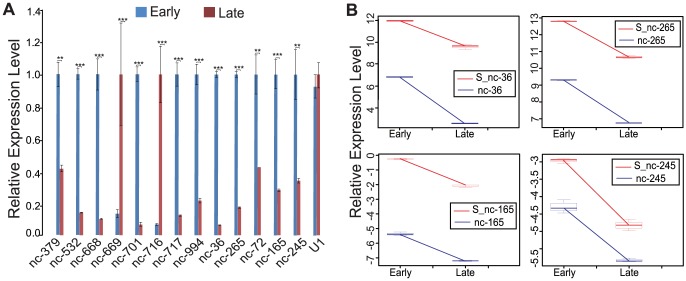
Expression profiles analysis of the novel is-ncRNAs. (A) qRT-PCR analysis of the expression of 13 novel is-ncRNAs during the intraerythrocytic developmental stage. U6 snRNA was used as an internal control to calculate the relative expression level (2^−ΔΔCt^) of each novel is-ncRNA. The known ncRNA U1 snRNA was used as reference controls. Statistically significant differences were determined using paired Student's T-test. **denotes p<0.01 and ***denotes p<0.001. (B) Relative expression analysis of the four novel antisense RNAs (blue) and their *cis*-encoded sense RNAs (red) during the intraerythrocytic developmental stages as determined by qRT-PCR. β-actin I was used as an internal control. The relative expression level was calculated as –ΔCt.

To investigate whether the expression of the novel is-ncRNAs was related to the morphology of the parasites, the differential expression of 13 novel is-ncRNA candidates were analysed by quantitative real-time PCR (qRT-PCR) in both the early (ring stage) and late stages (late trophozoites and schizonts). The analysis included nine intergenic is-ncRNAs and four antisense is-ncRNAs. Eleven of the thirteen is-ncRNAs showed significantly higher expression levels in the early stage than in the late stage; the remaining two intergenic is-ncRNAs, nc-669 and nc-716, were highly expressed in the late stage (Figure 5A). To confirm the expression differences between the time points, we selected the four antisense is-ncRNAs from the 13 aforementioned is-ncRNAs and determined the relationship between the expression levels of the antisense is-ncRNAs and their *cis*-encoded sense RNAs (Table 2) during the intraerythrocytic stage using qRT-PCR. All four pairs displayed positively co-regulated expression profiles during both early and late intraerythrocytic development (Figure 5B), consistent with most of the sense-antisense pairs analysed in a previous study [23].

**Table 2:** Details for the four antisense is-ncRNAs and their *cis*-encoded sense RNAs.

Antisense is-ncRNA	Chr	Start	End	Stra-nd	Sense RNA	Annotation of the sense RNA
nc-36	chr1	480698	480788	−	@S_nc-36	PF3D7_0112700:28S_rRNA
nc-165	chr4	617172	617246	+	S_nc-165	PF3D7_0413600:26S proteasome AAA-ATPase subunit RPT3, putative
nc-245	chr5	1069158	1069285	−	S_nc-245	PF3D7_0525700:Conserved *Plasmodium* protein, unknown function
nc-265	chr5	1295656	1295781	−	S_nc-265	PF3D7_0532000:28S_rRNA

@ S_nc denotes cis-encoded sense RNAs of the antisense is-ncRNAs.

Annotation of cis-encoded sense RNAs of all the novel antisense is-ncRNAs was listed in Table S5 in file S1.

## Discussion

In this study, a total of 1,198 novel *P. falciparum* is-ncRNA candidates were identified using a novel approach. We constructed an is-ncRNA library that was specifically depleted of rRNAs and U1–U6 RNAs and then sequenced this library using paired-end Illumina/Solexa paired-end sequencing. A total of 1,757 *P. falciparum* is-ncRNAs have been identified in this work and previous studies, greatly expanding our knowledge of the intermediate-size transcriptome of *P. falciparum*. These novel is-ncRNAs could not have been successfully identified without this strategy. The depletion of highly abundant rRNAs and U1–U6 RNAs during the construction of the is-ncRNA library allowed the efficient detection of novel is-ncRNAs within the intermediate-size RNA sample, as suggested by the fact that our data overlapped well (80.9%) with the is-ncRNAs (excluding tRNA, rRNAs and U1-U6 RNAs in this size range) found in PlasmoDB 10.0. Furthermore, the high-throughput sequencing technique has revolutionised the field of transcriptomics by facilitating RNA analysis through cDNA sequencing at unprecedented depths and massive scale compared to traditional cloning methods [37]. Hundreds of previously undetected transcripts in diverse tissues and organisms have been revealed using this new method [38]. Illumina/Solexa paired-end sequencing, which is one of the most widely used high-throughput sequencing technologies, can easily sequence homopolymeric regions [39], making this technique suitable for the very (A+T)-rich *Plasmodium* genome [17]. In contrast to the approximately 100-megabase *C. elegans* genome [40], which contains an estimated 7,000–10,000 intermediate-size transcripts [9], we predict that the 23-megabase *P. falciparum* genome [17] contains a total of 1,800–2,500 is-ncRNAs, requiring deeper mining of the novel is-ncRNAs.

The poor overlap with is-ncRNAs identified in previous studies may have occurred for one or more of the following reasons: (1) the size-fractionated RNA sample in the study that utilised the cloning method was treated with tobacco acid pyrophosphatase [23], while the RNA sample in the present study was not subjected to such a treatment and thus may have failed to identify is-ncRNAs with a 5′ cap structure; (2) our dedicated aim was to identify non-polyadenylated is-ncRNAs, while the is-ncRNAs detected in the study 2010 [29] were derived from a polyadenylated RNA sample; (3) the stringent depletion of highly abundant known ncRNAs in this study may have contributed to the loss of some transcripts; and (4) the tiling array may have a relatively high false positive rate due to cross-hybridisation [41].

In our work, the conservation of the novel *P. falciparum* is-ncRNA candidates, as reflected by their phastCons scores, was dependent on the genomic locations of the molecules. For instance, both the anti_mRNA exon is-ncRNAs and anti_ncRNA is-ncRNAs were shown to have a high degree of conservation, indicating the important role of these antisense RNAs during evolution, for functional sequences tend to be actively conserved throughout evolution [42]. In contrast, the intergenic is-ncRNAs were much less conserved among different *Plasmodium* species, which likely reflects the divergence within the genus *Plasmodium* [43] and may help explain why each *Plasmodium* species exhibits a restricted host range (e.g., primate parasites cannot infect rodent, bird or reptile hosts) [44]. The structure clustering in this work represents the first evidence that the intergenic is-ncRNAs of *P. falciparum* may be divided into eight classes, providing a framework with which to predict the targets of these is-ncRNAs. Additionally, two internal motifs, IM1 and IM2 identified in this study may facilitate further investigation of the molecular interaction between these novel is-ncRNAs and transcription factors. For example, novel is-ncRNAs with IM1 may be involved in the regulation of parasitic gene expression by interacting with serine/arginine-rich splicing factor 1, requiring further experiments to investigate.

In this study, significant differences in expression patterns were observed between the early and late stages of all nine intergenic and four antisense novel is-ncRNAs in *P. falciparum*, suggesting that the expression of the is-ncRNAs themselves is tightly regulated. The phenomenon that eleven out of the 13 randomly selected is-ncRNAs were more highly expressed in the early stage than in the late stage is quite interesting, since parasites undergo a fast development and remarkable differentiation within less than two days [45], the role of these is-ncRNAs during this stage obviously needs further investigations. Furthermore, the expression profiles of four pairs of antisense is-ncRNAs and their respective *cis*-encoded sense RNAs were shown to be positively co-regulated during the intraerythrocytic stage. Natural antisense transcripts (NATs) have been considered to negatively regulate the expression of their *cis*-encoded mRNA counterparts in both *P. falciparum* [46] and *Toxoplasma gondii* because a significant inverse correlation has been observed between the sense and antisense tag counts from single loci using SAGE technology [47]. However, the positive relationships between the NATs and their *cis*-encoded sense RNAs in *P. falciparum* were reported by Raabe *et al.* and Sims *et al.* [23], [48]. Our data were consistent with the latter results and also demonstrated that not all NATs inversely regulate the expression of their sense RNAs. Because NATs have been reported to form antisense-sense pairs that regulate transcription, mRNA stability, epigenetic silencing, and translation [49]–[52] in other organisms, it is clearly worthwhile to investigate the mechanism by which antisense is-ncRNAs function in *P. falciparum*.

Because the conventional RNAi pathway has not yet been identified in the *Plasmodium* [18], and no miRNAs have yet been demonstrated to exist in this parasite, is-ncRNAs may play critical roles in regulating gene expression, as shown by our study and other studies. Further, these novel is-ncRNA candidates are likely to regulate novel pathways during the development of the intraerythrocytic *P. falciparum* and this regulation may be unique to malaria parasites.

## Conclusions

Our work significantly increases the number of *P. falciparum* known is-ncRNAs and provides the first evidence that they may be classified into eight groups according to their secondary structures. The characterisation of the phase-specific expression of these is-ncRNAs implies that they may play critical roles in regulating gene expression during the development of the intraerythrocytic malaria parasite. Our study also highlights candidate avenues for future functional investigations, such as is-ncRNA overexpression or knockdown experiments, the validation of the interaction between is-ncRNAs and transcription factors and ubiquitin-protein ligase, and the mechanism by which antisense is-ncRNAs regulate and control parasite gene expression. A clear understanding of these topics will provide insights that may contribute to the eradication of this parasite.

## Materials and Methods

### Parasite culture and synchronisation

*P. falciparum* strain 3D7 was cultured in complete medium using the standard growth conditions originally described by Trager and Jensen [53], with minor modifications. Briefly, the parasites were cultured with human red blood cells and RPMI Medium 1640 at 37°C in petri dishes (5% haematocrit). The medium was supplemented with 25 mM HEPES, 10 mg/L glucose, 0.5% Albumax II, 0.3 g/L L-glutamine, 0.015% hypoxanthine, 0.125 g/L gentamicin, and 25 mM sodium bicarbonate. To prepare is-ncRNA-specific library for Illumina/Solexa paired-end sequencing, about 30 petri dishes (90 mm in diameter) of the parasite culture at mixed intraerythrocytic stage were collected for total RNA extraction, with 600 µl red blood cells pellet at a parasitemia between 5 to 10% in each petri dish. To enrich for early-stage parasites, the cultures were synchronised twice with a 5% sorbitol solution treatment [54]. To enrich for late-stage parasites, a discontinuous gradient centrifugation in Percoll (Pharmacia) containing 5% D-sorbitol was performed as previously described [55], [56]. The Percoll gradient consisted of three layers (90%, 70% and 40%); the 40–70% interface, containing mostly late trophozoites and schizonts, was collected, washed, and prepared for total RNA extraction.

### Total RNA extraction and preparation

To isolate the parasites, harvested cultures were treated with 0.1% saponin, and the released parasites were pelleted by centrifugation and washed twice with ice-cold phosphate-buffered saline (PBS). Total RNA was extracted using the TRIzol Reagent (Invitrogen) according to the manufacturer's instructions. The RNA was quantified with a NanoDrop 1000 Spectrophotometer (Thermo Scientific), and the RNA quality was confirmed using 6% denaturing urea polyacrylamide gel electrophoresis (PAGE).

### Illumina/Solexa paired-end sequencing library preparation

The is-ncRNA-specific library was constructed as previously described [57], with modifications. Briefly, 200 µg of DNAse I-treated RNA was size fractionated via anion exchange chromatography on an RNA/DNA Midi Tip (QIAGEN) according to the manufacturer's instructions. RNAs that ranged in size from 50 to 500 nt were pooled. RNA sizes were estimated using the RiboRuler™ Low-Range RNA Ladder (Fermentas). Highly abundant 5S and 5.8S rRNAs, U3 snoRNA, and U1–U6 snRNAs were removed using the Ambion MICROBExpress kit (Ambion) with specifically designed Capture Oligonucleotides (Table S6 in file S1). The is-ncRNA sample was separated into a 50–200 nt fraction and 200–500 nt fraction on a 6% denaturing urea PAGE gel. After the gel fragments were excised and the RNA was purified, the 50–200 nt fraction was dephosphorylated, ligated to a 3′ adaptor (3′AD, 5-p-GAUCGGAAGAGCGGUUCAGCAGGAAUGCCGAG-3) and reverse transcribed with a universal primer (3′RT, CTCGGCTTTCCTGCTGTTCCG). The 200–500 nt fraction were directly reverse transcribed with a random primer (5-CTCGGCTTTCCTGCTGTTCCGNNNNNN-3). Another adaptor (5-ACACTCTTTCCCTACACGACGCTCTTCCGATCT-3) was ligated to the 3′ends of the two cDNA samples. PCR was performed, and the amplified products were sequenced on a standard Illumina/Solexa paired-end sequencing platform.

### Computational analysis

The 5′ and 3′ adaptors in the raw paired-end sequencing data were removed, and reads with abnormal adaptors or inserted sequences shorter than 30 nt were filtered. For all the remaining clean reads, the first 51 bp of each read were retained and were mapped to PlasmoDB 10.0 (http://plasmodb.org/common/downloads/release-10.0/Pfalciparum3D7/) using Bowtie [28] (-a -v 1 –best –strata -x 1000 -y). Only reads (51 bp) containing no more than one mismatch were considered as qualified reads and were used for further analysis. The qualified paired-end reads with at least one nt overlapping were clustered into contigs, and “wig” profiles were generated for visualisation in the Integrated Genome Brower [58]. Genome annotation and sequence data was downloaded from PlasmoDB 10.0. The phastCons score data for conservation analysis was downloaded from the UCSC Malaria Genome Browser (http://areslab.ucsc.edu/data_downloads/) after sequences of the contigs were mapped to the UCSC Malaria Genome (2007). Additionally, ncRNAs that had been reported or analysed in five previous studies [23], [24], [26], [27], [29] but not annotated in PlasmoDB 10.0 were collected and referred to as “reported ncRNAs”. The obtained contigs that overlapped with these reported ncRNAs were removed (filtering criterion: BLAST e<10E-5). Conservation analysis was then performed using phastCons scores. The snoGPS [31] and snoReport [32] programmes were used to test whether the is-ncRNA candidates were likely to be snoRNAs. Other potential homologs of known ncRNAs were identified via sequence alignment with the 1,371 ncRNA families in the Rfam database [33] using the HMMER3 (http://hmmer.janelia.org/) server. A structure-based analysis of the is-ncRNAs was performed using LocARNA-based clustering [36], and the tree was generated with iTOL [59]. Sequence motif detection was performed with MEME [60].

### RT-PCR confirmation of novel is-ncRNAs

RT-PCR was performed using the RevertAid™ First-Strand cDNA Synthesis Kit (Fermentas) according to the manufacturer's recommendations. Specific primers (see Table S7 in file S1) were designed based on the sequence of each novel is-ncRNA. The PCR was carried out with an initial denaturation at 94°C for 5 min; followed by 35 cycles of 94°C for 30 s, an appropriate annealing temperature (ranging from 47.5°C to 55°C) for 40 s, and 72°C for 45 s; and a final extension for 10 minutes at 72°C. The PCR products were electrophoresed on PAGE gels, and the sizes of the PCR products were estimated using a 50-bp DNA ladder. Negative controls, including RT reactions lacking either DNase I-treated RNA or reverse transcriptase, were performed simultaneously to demonstrate the accuracy and specificity of the RT-PCR.

### Northern blotting analysis

RNA probes were labelled and transcribed in vitro with SP6 RNA polymerase (Promega) and dig-11-UTP (Roche). Total RNA samples (10 µg) were resolved by 7M urea 6% denaturing PAGE gel electrophoresis and transferred onto a nylon membrane (Hybond-N+, GE Healthcare) by capillary transfer in 20×SSC for at least 6 hours. The RNA sample on the membrane was then fixed by UV crosslinking. Next, the membrane was rinsed briefly in double distilled water. The membranes were subsequently hybridised with the DIG-labelled RNA probes in Hyb buffer (Roche) at 55°C overnight. Northern blotting was performed according to the manufacturer's instructions. After hybridisation, the blots were treated with Washing and Blocking Buffer (Roche), immunologically detected with anti-digoxigenin-AP, and equilibrated in detection buffer. Finally, the blots were incubated with the chemiluminescent substrate CDP-star (Roche) and exposed to Kodak film. A DIG-labelled U5 RNA probe was used as an internal control. The primers used for DIG-labelled RNA probe transcription are listed in Table S8 in file S1.

### Quantitative RT-PCR analysis of novel is-ncRNAs

DNase I-treated total RNA (300 ng) from the early and late intraerythrocytic stages of *P. falciparum* 3D7 was reverse transcribed. qRT-PCR was performed with 1∶10 diluted cDNAs, a StepOnePlus™ Real-Time PCR System (Applied Biosystems) and TransStart Green qPCR SuperMix (Transgen) under the following cycling conditions: 95°C for 5 min; 40 cycles of 95°C for 15 s, 55°C for 20 s and 72°C for 20 s; and a final extension at 72°C for 5 min. A melting curve was generated as follows: 95°C for 15 s, 55°C for 1 min followed by a temperature gradient with a ramp rate of 0.5°C/s, and a final denaturation at 95°C for 15 s. The obtained products were also evaluated on a 6% denaturing urea PAGE gel. For relative quantification, a house-keeping gene (Pf-β-actin I or U6 snRNA, as appropriate) was used as an internal control. For the intergenic is-ncRNAs, qRT-PCR analysis was also performed on a Rotor-Gene Q apparatus (QIAGEN) using the supplied software and TransScript II Green One-step qRT-PCR SuperMix (Transgen) according to the manufacturer's instructions. The primers used in these experiments are listed in Table S9 in file S1 and the Ct values found for the real time experiment are presented in Table S10 in file S1.

## GEO accession number

The data obtained from the Illumina/Solexa paired-end sequencing in this study was deposited in the Gene Expression Omnibus database at NCBI (http://www.ncbi.nlm.nih.gov/geo/). The accession number for GEO is GSE45172.

## Supporting Information

File S1 **Table S1. The 1,198 novel is-ncRNA contigs assembled from the Illumina/Solexa paired-end sequencing. Table S2. Novel is-ncRNAs with predicted structures. Table S3. Novel is-ncRNAs with internal motifs (IM1 and IM2). Table S4. The 60 novel is-ncRNAs confirmed by RT-PCR. Table S5. Annotations of the** ***cis*** **-encoded sense RNAs of the novel antisense RNAs. Table S6. Oligos used to remove known ncRNAs. Table S7. Primers used for RT-PCR. Table S8. Oligos used to obtain RNA probes in northern blotting assays. Table S9. Primers used for qRT-PCR. Table S10. Ct values found for the qRT-PCR.**
(XLS)

File S2 **Figure S1. Internal motifs (IM1 and IM2) of the novel intergenic is-ncRNAs and similar RNA motifs in** ***Homo sapiens*** **. Figure S2. RT-PCR confirmation of additional 50 novel is-ncRNAs.** Each is-ncRNA is represented by three adjacent lanes; from left to right, these lanes include DNase-treated RNA RT-PCR (“+”), RT-PCR with no RNA template (left, “-”, negative control) and RT without reverse transcriptase (right, “-”, negative control). “M” indicates the 50 bp DNA ladder. “Length” indicates the expected sizes of the is-ncRNAs based on the Illumina/Solexa paired-end sequencing assembly data.
(PDF)
