# Supplementary figures and images for: Deep Profiling of the Novel Intermediate-Size Noncoding RNAs in Intraerythrocytic Plasmodium falciparum
Source: PLoS One. 2014 Apr 8;9(4):e92946. doi: 10.1371/journal.pone.0092946 (PMC3979661; doi:10.1371/journal.pone.0092946)

$p=0.003$

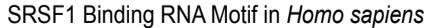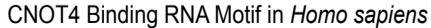

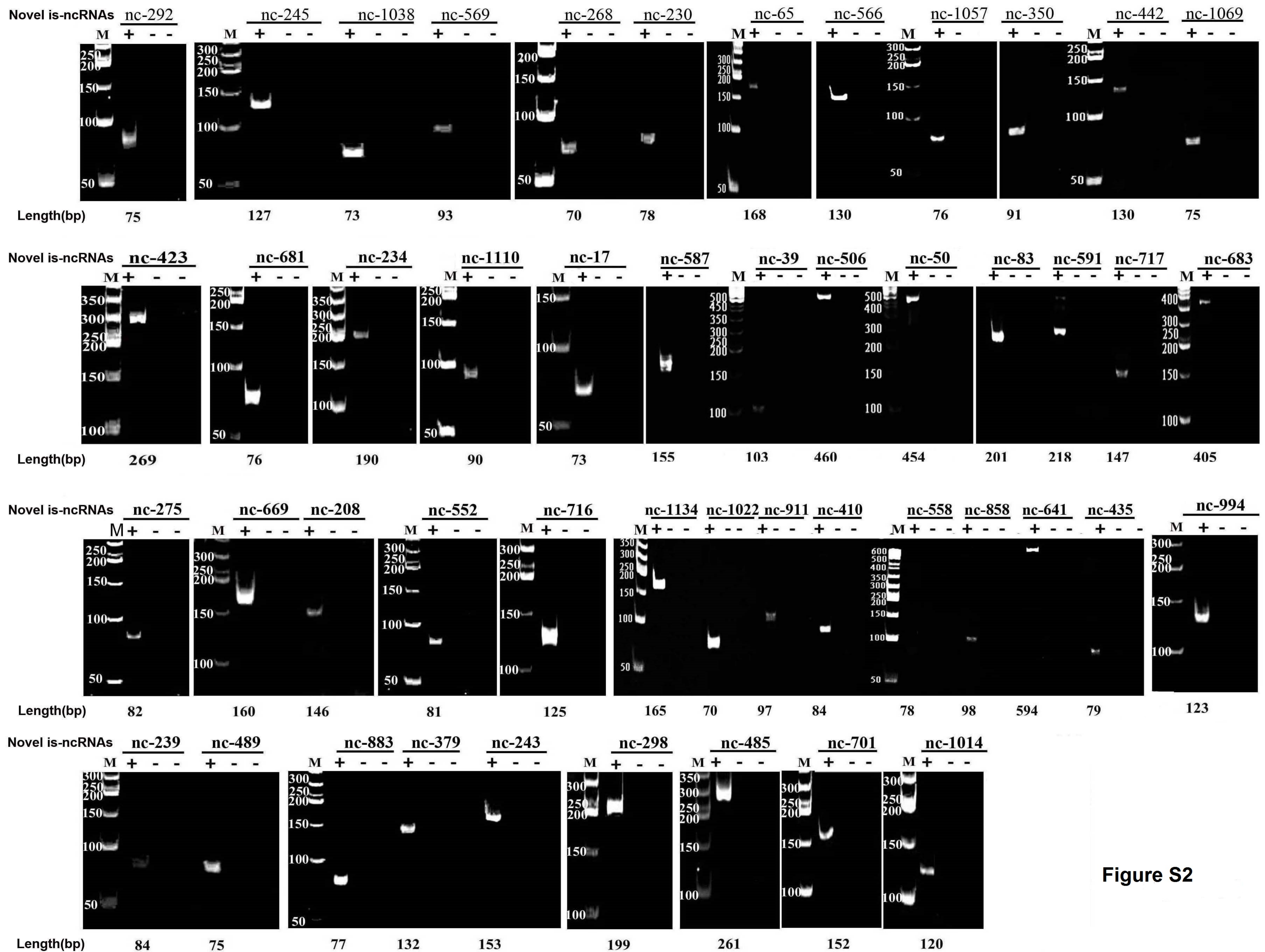

**Figure S2**

Supplement: File S2 — Figure S1. Internal motifs (IM1 and IM2) of the novel intergenic is-ncRNAs and similar RNA motifs in Homo sapiens . Figure S2. RT-PCR confirmation of additional 50 novel is-ncRNAs. Each is-ncRNA is represented by three adjacent lanes; from left to right, these lanes include DNase-treated RNA RT-PCR (“+”), RT-PCR with no RNA template (left, “-”, negative control) and RT without reverse transcriptase (right, “-”, negative control). “M” indicates the 50 bp DNA ladder. “Length” indicates the expected sizes of the is-ncRNAs based on the Illumina/Solexa paired-end sequencing assembly data. (PDF) [file pone.0092946.s002.pdf]
